# A minimal i-motif stabilized by minor groove G:T:G:T tetrads

**DOI:** 10.1093/nar/gks911

**Published:** 2012-10-05

**Authors:** Núria Escaja, Júlia Viladoms, Miguel Garavís, Alfredo Villasante, Enrique Pedroso, Carlos González

**Affiliations:** ^1^Departament de Química Orgànica and IBUB, Universitat de Barcelona, Martí i Franquès 1-11, 08028 Barcelona, ^2^Instituto de Química Física Rocasolano, CSIC, Serrano 119, 28006 Madrid and ^3^Centro de Biología Molecular “Severo Ochoa” (CSIC-UAM), Universidad Autónoma de Madrid, Nicolás Cabrera 1, 28049 Madrid, Spain

## Abstract

The repetitive DNA sequences found at telomeres and centromeres play a crucial role in the structure and function of eukaryotic chromosomes. This role may be related to the tendency observed in many repetitive DNAs to adopt non-canonical structures. Although there is an increasing recognition of the importance of DNA quadruplexes in chromosome biology, the co-existence of different quadruplex-forming elements in the same DNA structure is still a matter of debate. Here we report the structural study of the oligonucleotide d(TCGTTTCGT) and its cyclic analog d<pTCGTTTCGTT>. Both sequences form dimeric quadruplex structures consisting of a minimal i-motif capped, at both ends, by a slipped minor groove-aligned G:T:G:T tetrad. These mini i-motifs, which do not exhibit the characteristic CD spectra of other i-motif structures, can be observed at neutral pH, although they are more stable under acidic conditions. This finding is particularly relevant since these oligonucleotide sequences do not contain contiguous cytosines. Importantly, these structures resemble the loop moiety adopted by an 11-nucleotide fragment of the conserved centromeric protein B (CENP-B) box motif, which is the binding site for the CENP-B.

## INTRODUCTION

Since the description of the left-handed Z-DNA in 1979, a great variety of alternative non-B DNA structures has been characterized (1). Many of these non-canonical secondary structures, such as cruciforms (2,3), triplexes (4), quadruplexes (5) or slipped structures (6) are associated with specific repetitive patterns that, interestingly, are found in functionally important genomic regions (7). Although these unusual non-B DNA structures seem to be functional chromosomal elements, they also induce genetic instability resulting in predisposition to diseases (8,9).

Genomic DNA sequences containing runs of guanines or cytosines have the potential to form G-quadruplexes or i-motif structures, respectively. G-quadruplexes are the most extensively studied (10). The *in vivo* evidence of their existence at telomeres (11) and gene promoters (12,13), their role in controlling different biological processes (14,15), their potential therapeutic applications (16,17) and their interesting uses in supramolecular chemistry (18) have converted G-quadruplexes in primary research targets.

The so-called i-motif is a four-stranded intercalated structure formed by the association of two parallel-stranded duplex connected by hemi-protonated C:C^+^ base pairs (Supplementary Figure S1B). The two duplexes are intercalated in opposite orientations. Since i-motif formation requires protonation of cytosines (19), these structures are more stable at acidic pH, although they can also be detected at nearly neutral pH. I-motif structures can also be stabilized by external agents, such as molecular crowding agents (20), single-walled carbon nanotubes (21), or site-specific incorporation of porphyrin moieties (22). Several i-motif structures have been found in oligonucleotides from centromeric (23,24) and telomeric (25) repetitive sequences. Dynamics (26) and folding studies (27) on the i-motifs of these repetitive sequences have been carried out recently. Moreover, studies have revealed the formation of i-motif secondary structures in the promoter region of oncogenes such as bcl-2, c-Myc, VEGF and RET (28). In addition, i-motif structures are utilized in nanotechnology (29). Recent applications in this field include the design of DNA-based devices to monitor intracellular pH (30).

In addition to G-quadruplexes and i-motifs, four-stranded structures can also be formed by both major and minor groove-aligned tetrads (Supplementary Figure S1), although they remain much less understood. All major groove tetrads reported so far have been found within the scaffold of G-quadruplexes (31–37). The quadruplexes formed by minor groove tetrads have been studied in our and other laboratories (38,39), and it has been found that these tetrads are rather general. They are principally formed by different arrangements of canonical G:C and A:T base pairs (40–43). Some of them have been observed in folded-back linear oligonucleotides (44), although they are better observed in cyclic analogs due to their higher stability and the absence of competing Watson–Crick duplex structures. The main distinctive features of these minor groove tetrads are the mutual inclination of around 30°–40° between base pairs and the close proximity of the sugar-phosphate backbone of the strands containing the minor groove interacting bases.

Recently, we found that minor groove tetrads can also be formed by G:T mismatched base pairs (45), and we noticed that a similar minor groove G:T:G:T tetrad had previously been observed in the solution structures of oligonucleotides from the pyrimidine-rich strand of the centromeric protein B (CENP-B) box sequence (23,46,47). The CENP-B box is the conserved binding site for the centromeric protein CENP-B in mammalian centromeric satellite DNA (48,49). The conservation of the CENP-B box in mammalian species indicates that this sequence has been selected to play a role in mammalian centromere formation, but this role remains to be established.

At acidic pH, the oligonucleotides from the relatively pyrimidine-rich CENP-B box strand d(CCCGTTTCC), d(TCCCGTTTCCA) and the full d(TCCCGTTTCCAACGAAG) fold and dimerize in solution to form a four-stranded intercalated motif with five hemi-protonated C:C^+^ base pairs (23). A pair of GTTT loops are at the same end of the double hairpin, and these two loops interact with each other through a minor groove-aligned slipped G:T:G:T tetrad. Interestingly, the GTTT sequence involved in these interactions corresponds to a highly conserved region of the CENP-B box.

**Figure 1.**
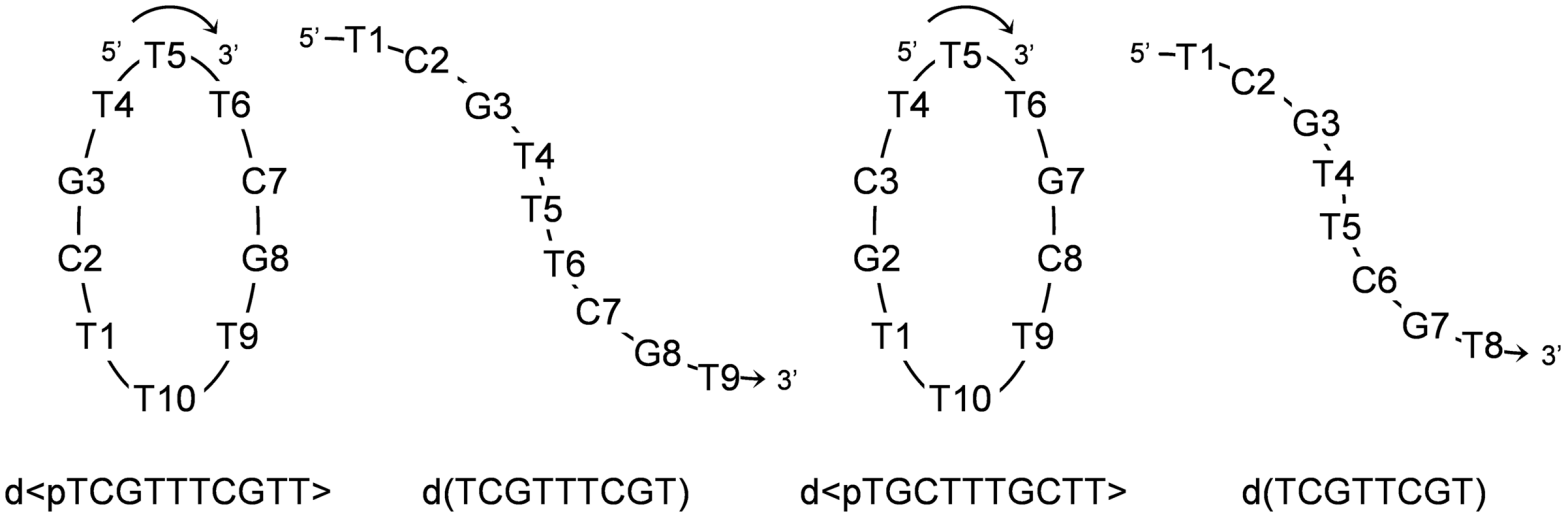
Scheme and numbering of the oligonucleotide sequences.

To explore the connections between minor groove tetrads and i-motif structures, we decided to study whether minor groove-aligned G:T:G:T tetrads can stabilize a minimal i-motif. To this end, we have studied the solution structure and stability of the oligonucleotide d(TCGTTTCGT) and its cyclic analog d<pTCGTTTCGTT> (Figure 1) by CD, NMR and restrained molecular dynamics. In both cases, their structures show a minimal i-motif, consisting of two hemi-protonated C:C^+^ base pairs, and a slipped minor groove G:T:G:T tetrad at both ends of the C:C^+^ base stacks. These tetrads are capped by stacking interactions of two thymine residues of the loop, resulting in a quite unusual compact structure. The analysis of two sequence-related oligonucleotides, d(TCGTTCGT) and d<pTGCTTTGCTT>, indicates that the minor groove G:T:G:T tetrad is essential to stabilize the mini i-motif.

## MATERIALS AND METHODS

### Experimental details

The cyclic oligonucleotide was synthesized as reported by Alazzouzi *et al.* (50). The linear oligonucleotides were synthesized by standard phosphoramidite chemistry. Samples for NMR experiments were dissolved (in Na^+^ form) in either D_2_O or 9:1 H_2_O/D_2_O. Most experiments were carried out in 25 mM sodium phosphate buffer, 100 mM NaCl. In some cases, 10 mM MgCl_2_ was added. pH was adjusted by adding small amounts of concentrated HCl. No change in the NMR spectra was observed upon different annealing protocols. All NMR spectra were acquired in Bruker spectrometers operating at 600 MHz and 800 MHz, equipped with cryoprobes and processed with the TOPSPIN software. In the experiments in D_2_O, presaturation was used to suppress the residual H_2_O signal. A jump-and-return pulse sequence was employed to observe the rapidly exchanging protons in 1D H_2_O experiments. NOESY spectra in D_2_O and 9:1 H_2_O/D_2_O were acquired with mixing times ranging from 100 ms to 300 ms. TOCSY spectra were recorded with the standard MLEV-17 spin-lock sequence and a mixing time of 80 ms. In most of the experiments in H_2_O, water suppression was achieved by including a WATERGATE (51) module in the pulse sequence prior to acquisition. The spectral analysis program SPARKY (52) was used for semiautomatic assignment of the NOESY cross-peaks and quantitative evaluation of the NOE intensities.

Circular dichroism spectra at different temperatures were recorded on a Jasco J-810 spectropolarimeter fitted with a thermostated cell holder. CD spectra were recorded in 25 mM sodium phosphate buffer, pH 7, with 100 mM NaCl and 10 mM MgCl_2_ or 20 mM sodium acetate buffer, pH 4.5, with 200 mM NaCl. For melting experiments, the samples were initially heated at 90°C for 5 min, and slowly allowed to cool to room temperature and stored at 4°C until use. CD melting curves were recorded at the wavelength of the larger positive band, 262 nm, with a heating rate of 0.5°C·min^−^^1^. For pH titration, NaOH was added to samples dissolved in 20 mM acetate buffer, initially at pH 4.5, containing 200 mM NaCl.

For extracting thermodynamic parameters, the CD melting curves were converted into representations of the molar fraction of structured species against temperature, which allowed to obtain the equilibrium constant of the process at each temperature. The plotting of ln*K* as a function of 1/*T* displayed a linear dependence that allowed to obtain the thermodynamic parameters by fitting the points to the function ln*K* =ΔS°/R – (ΔH°/R)·1/T with the program OriginPro 7.5. Propagation error methods were applied to calculate the associated errors to the obtained thermodynamic parameters.

### NMR constraints

Initial calculations were performed with qualitative distance constraints (classified as 3, 4 or 5 Å) and the resulting structures were then refined by employing more accurate distance constraints obtained from a complete relaxation matrix analysis with the program MARDIGRAS (53). Error bounds in the interprotonic distances were estimated by carrying out several MARDIGRAS calculations with different initial models, mixing times and correlation times, as described in previous works. In addition to these experimentally derived constraints, hydrogen bond restraints were used. Target values for distances and angles related to hydrogen bonds were set to values obtained from crystallographic data in related structures (54).

### Structure determination

Structures were calculated with the program DYANA 1.4 (55) and further refined with the SANDER module of the molecular dynamics package AMBER 7.0 (56). Initial DYANA calculations were carried out on the basis of qualitative distance constraints. The resulting structures were used as initial models in the complete relaxation matrix calculations to obtain accurate distance constraints, as described in the previous paragraph. These structures were taken as starting points for the AMBER refinement, consisting of an annealing protocol *in vacuo*, followed by long trajectories where explicit solvent molecules were included and using the Particle Mesh Ewald method to evaluate long-range electrostatic interactions. The specific protocols for these calculations have been described elsewhere (57). The AMBER-98 force field (58) was used to describe the DNA, and the TIP3P model was used to simulate water molecules (59). Analysis of the representative structures as well as the MD trajectories was carried out with the programs Curves V5.1 (60) and MOLMOL (61).

## RESULTS

### d(TCGTTTCGT) and d<pTCGTTTCGTT> adopt dimeric structures with hemi-protonated C:C^+^ base pairs

#### CD spectra

**Figure 2.**
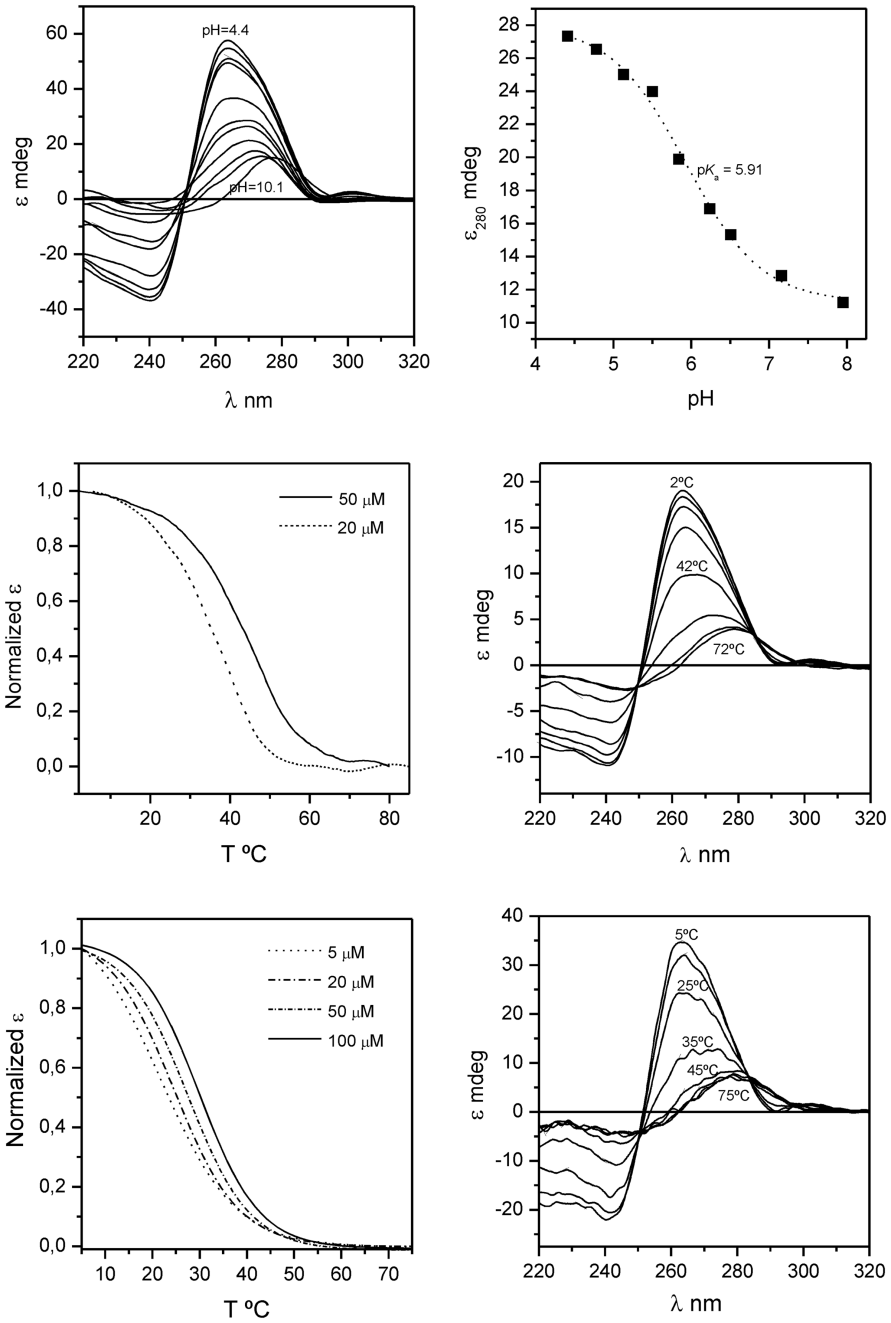
*Top*: series of CD spectra of d<pTCGTTTCGTT> at different pH (left) and pH titration (right), [oligonucleotide] = 20 µM, T = 5°C. *Middle*: CD melting curves of d<pTCGTTTCGTT> at different oligonucleotide concentration (left) and series of CD spectra at different temperature, [oligonucleotide] = 50 µM (right). *Bottom*: CD melting curves of d(TCGTTTCGT) at different oligonucleotide concentration (left) and series of CD spectra at different temperature, [oligonucleotide] = 50 µM (right). Buffer conditions: 20 mM AcONa, pH 4.5, 200 mM NaCl.

CD spectra of d(TCGTTTCGT) and d<pTCGTTTCGTT> exhibit strong dependence on temperature and oligonucleotide concentration. For d<pTCGTTTCGTT> (Figure 2, middle) increasing oligonucleotide concentration from 20 µM to 50 µM raises the *T*_m_ value from 38°C to 42°C, indicating a concentration dependent melting behavior, characteristic of multimeric structures. In addition, the CD spectra also show a strong dependence on pH (Figure 2, top). The CD spectrum at pH 4.5 and low temperature exhibits a small negative band at 240 nm, a large positive band at 262 nm and a small positive band at 302 nm. The thermal unfolding transition followed by CD shows a bathochromic shift of the band at 262 nm, accompanied by a progressive disappearance of all the three bands. Similar changes in the CD spectra are observed upon pH titration, indicating a pH dependent denaturation process. The midpoint pH of this transition provides an apparent p*K*_a_ value for the overall structure of 5.9. Different counter-ions (Na^+^, Mg^2+^) do not affect significantly the global shape of the CD spectra at 5°C, indicating a similar degree of structuration (data not shown), although increasing ionic strenght (i.e. by addition of Mg^2+^) enhances the thermal stability of the dimeric species. An analog behavior is observed in the CD spectra of d(TCGTTTCGT) (Figure 2, bottom). As expected, the CD spectra indicate a low degree of structuration under very low salt concentration (data not shown).

The CD spectra of d(TCGTTTCGT) and d<pTCGTTTCGTT> at acidic pH do not exhibit the strong positive band at around 285 nm and the weaker negative band at around 254 nm, characteristic of an i-motif structure (62). Except for the small band at 302 nm, the CD spectra resemble that observed for the dimeric quadruplex structures stabilized by slipped minor groove tetrads (41,45). The small band around 300 nm arising at low pH has been observed in cytosine protonated species (63).

#### NMR

**Figure 3.**
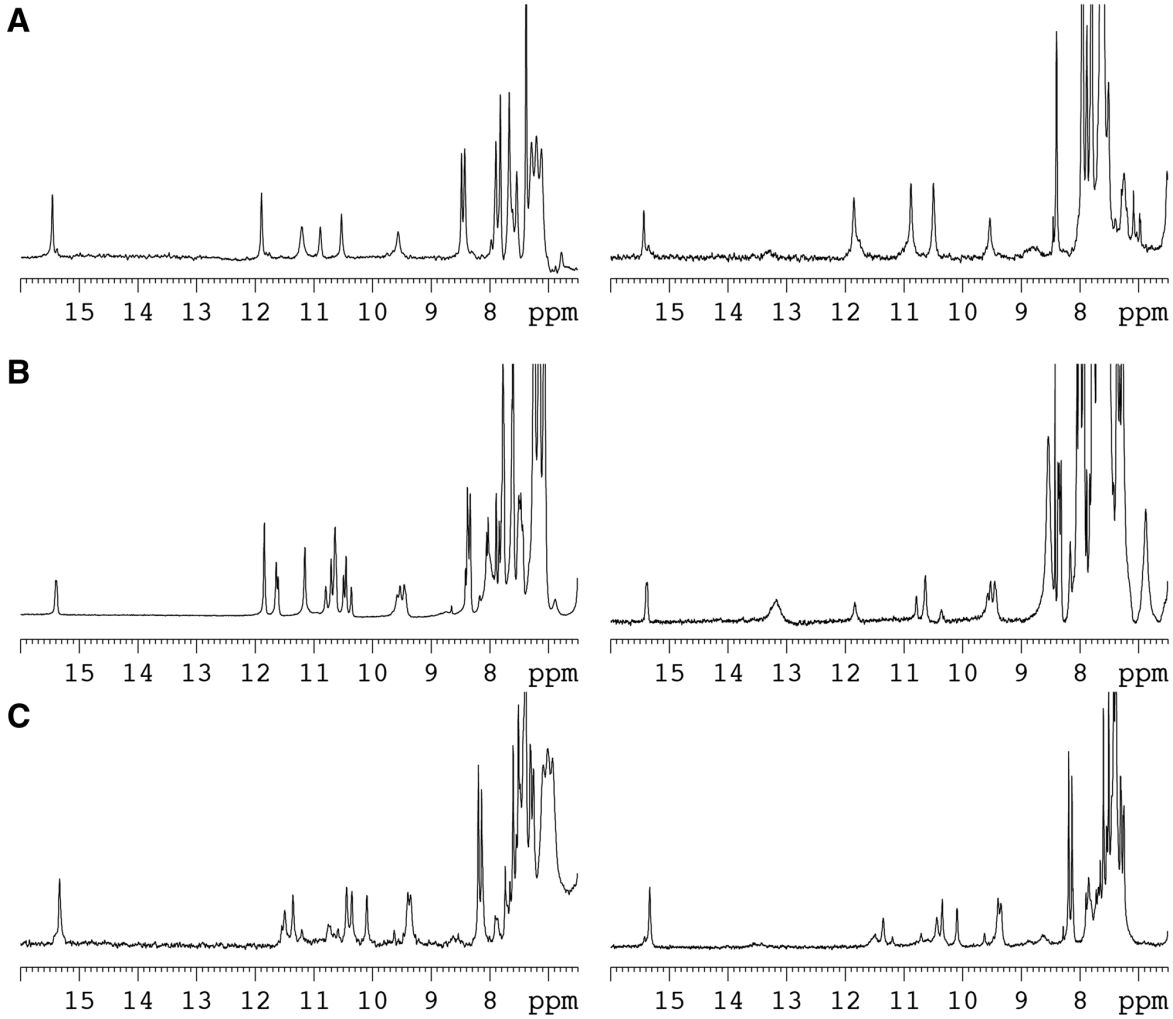
NMR spectra of d<pTCGTTTCGTT> (**A**), d(TCGTTTCGT) (**B**) and d(TCGTTCGT) (**C**) in H_2_O/D_2_O 9:1 at T = 5°C in phosphate buffer, 100 mM NaCl, oligonucleotide concentrations around 0.5 mM. Left panel spectra at pH 5; Right panel spectra at pH 7.

The ^1^H-NMR spectra of d(TCGTTTCGT) and d<pTCGTTTCGTT> at 5°C depend strongly on experimental conditions (Figure 3). At oligonucleotide concentrations around 0.5 mM and acidic pH, the exchangeable protons spectra exhibit signals characteristic of non-canonical base pairs. The signal at 15.4 ppm is distinctive of cytosine imino protons in hemi-protonated C:C^+^ base pairs (as well as the signals at 9.52 ppm for their amino protons) (64). The sharp signals at 11.85 and 10.85 ppm can be attributed to mismatched G:T base pairs. Interestingly, most of these signals are also observed at neutral pH. Signals of exchangeable protons disappear completely when the spectra is recorded at lower oligonucleotide concentration (Supplementary Figure S2). The pH dependence of the NMR spectra indicates the formation of hemi-protonated C:C^+^ base pairs. The changes on NMR spectra with temperature and oligonucleotide concentration are consistent with dimeric structures (Supplementary Figure S3). The pH dependent formation of these structures and their molecularity were confirmed by native gel electrophoresis experiments (Supplementary Figure S4). NMR melting experiments indicate that the equilibrium between the dimer and the unfolded single-stranded species is slow in the NMR time scale.

**Figure 4.**
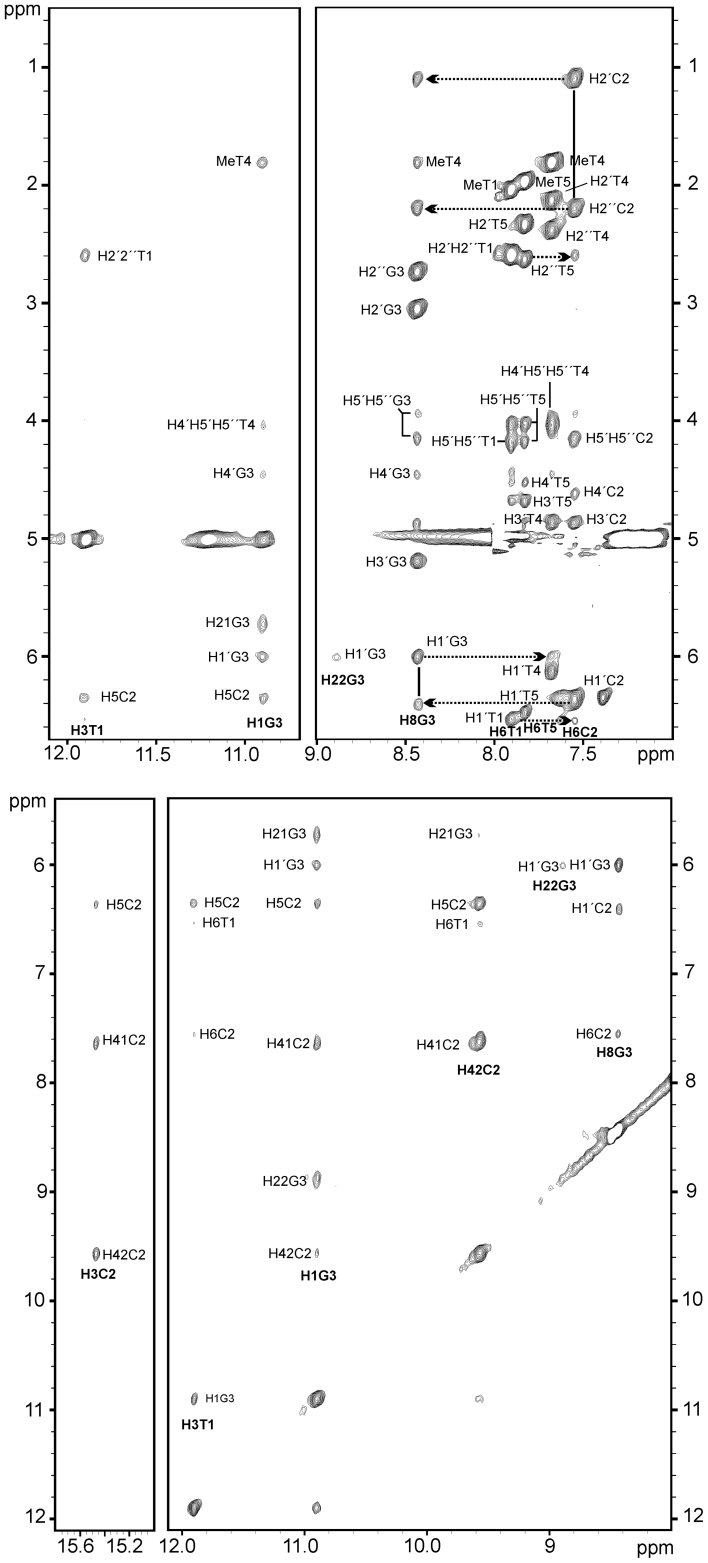
Regions of the NOESY spectra (*t*_m_ = 300 ms) of d<pTCGTTTCGTT> in H_2_O/D_2_O 9:1. *Top*: imino-sugar protons region showing cross-peaks involving guanine (G3) and thymine (T1) residues forming G:T base pair (left) and Ar-sugar protons region showing the sequential connectivities T1→C2→G3→T4 (right). *Bottom*: imino region of protonated cytosine (C2) forming hemi-protonated C:C^+^ base pair (left) and imino and amino region showing connectivities involving imino protons of thymine (T1) and guanine (G3) residues and amino protons of guanine (G3) and cytosine (C2) residues (right). *Conditions*: 0.5 mM oligonucleotide concentration, 25 mM phosphate buffer, 100 mM NaCl, T = 5°C, pH 5.1. Cross-peaks of d<pTCGTTTCGTT> are labeled according to the spin systems numbering shown in Figure 1.

In the case of the cyclic decamer, the sequence is repetitive and only signals corresponding to five distinct residues are expected for a monomeric species. Interestingly, the number of signals in the NMR spectra of the high concentration species of d<pTCGTTTCGTT> also corresponds with 5 nt. This fact clearly indicates that its structure is a homodimer with a 2-fold symmetry. The NMR spectra of d(TCGTTTCGT) exhibit very similar features. However, this sequence is not completely repetitive, and NMR signals are not degenerated (Figure 4 and Supplementary Figures S5 and S7). Complete spectral assignment in this case is very problematical and, consequently, we decided to focus on the structural determination of the cyclic analog.

### NMR assignment of d<pTCGTTTCGTT>

Sequential assignment of exchangeable and non-exchangeable protons was conducted following standard methods. As previously mentioned, only five spin systems were observed in the NMR spectra at pH 5. Residues 1-5 are equivalent to residues 6-10 within the same sub-unit, and to the residues 11–15 and 16-20, for the opposite sub-unit in the dimer. In this section we will use the numbering 1-5 for the discussion of the NMR assignment, indicating inside brackets for the equivalent residues in each sub-unit. The numbering 1-20 will be used for describing the structure.

#### Non-exchangeable protons

All the resonances were identified although none of the H5′ and H5″ protons could be stereo specifically assigned. Clear sugar-base sequential connectivities H8-H1′/H2′/H2″ were observed for the residues C2(7)→G3(8) (Figure 4). An additional H6C2(7)-H8G3(8) cross-peak was also observed for these stacked residues. Thymine residues could be distinguished on the basis of some cross-peaks with guanine and cytosine residues. Weak sequential cross-peaks were observed between thymine H1′/H2′/H2″ protons and the aromatic proton of C2(7), consequently this thymine was assigned as T1(6). T4(9) was identified by the weak sequential cross-peak H1′G3(8)-H6T4(9) and additional connectivities between its methyl protons and the H8 and H1′ protons of G3(8). No sequential connectivity involving T5(10) was found, but some cross-peaks were observed between H4′/H5′/H5″ protons of T5 and H6 of T1 (Figure 4) and between H5′/H5″ protons of T5 and H2′ of T4. A complete map of connectivity and the list of assignments are given in Supplementary Figure S8 and Supplementary Table S1, respectively.

All the residues show medium or weak intranucleotide H1′-base NOEs, indicative of an *anti* conformation. The intensity of the intranucleotide H3′-base NOEs denote a C2′-*endo* sugar conformation (*S*-type) for all the residues. For the G2 residue the H3′-base cross-peak is not as weak as for the rest of the residues but it shows a weak H2″-H4′ cross-peak, that is typical for an *S*-type sugar.

#### Exchangeable protons

**Figure 5.**
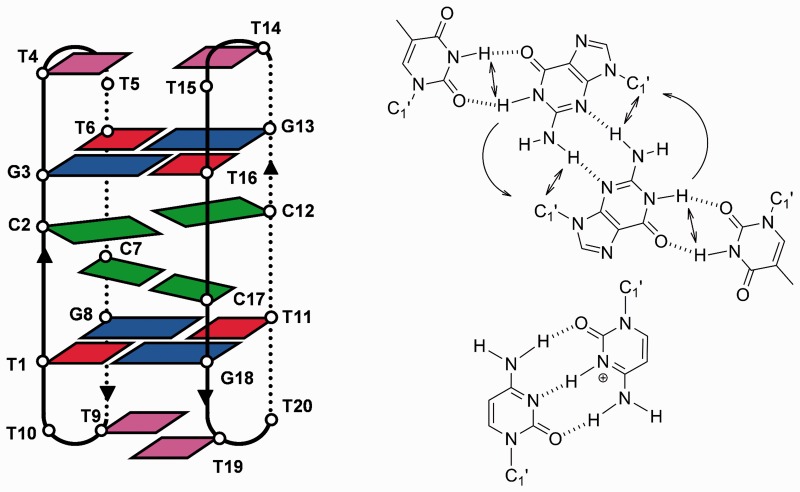
*Left*: schematic representation of the dimeric structure of d<pTCGTTTCGTT>. *Right*: G:T:G:T minor groove tetrad showing the observed connectivities in the NMR spectra, and C:C^+^ base pair.

Five imino proton signals were observed in the 1D ^1^H-NMR spectrum at pH 5 and low temperature (Figure 3A). These signals could be detected at temperatures close to the *T*_m_, indicating a compact structure with protected exchangeable protons. The imino signal at 15.42 ppm corresponds to C2(7), forming hemi-protonated C:C^+^ base pairs. As expected, this signal integrates approximately half of the signal of the other imino peaks. The remaining imino protons resonate between 10.0 ppm and 12 ppm. The NOE cross-peak pattern involving two of these protons (10.85 ppm and 11.85 ppm) clearly indicates the formation of a wobble G:T base pair. The other peaks correspond to unpaired thymines. A number of sequential cross-peaks with C2(7) permitted the assignment of the G:T base pair to residues G3(8) and T6(1) (Figure 4). Interestingly, imino and amino protons of G3(8) showed intense cross-peaks with their own H1’ protons. Since the intraresidual distance is too large, we conclude that this is an intermolecular NOE, indicative of a G:G interaction. Also the guanine amino chemical shifts suggest that they are involved in hydrogen bond formation. These experimental data are consistent with an intermolecular interaction of two guanines across the minor groove involving hydrogen bonding of one amino proton with the N3 of the opposite guanine. The resulting arrangement is a G:T:G:T tetrad, probably similar to minor groove G:T:G:T tetrads previously reported in the structures of some cyclic octamers (45) (Figure 5, right). In addition, other significant cross-peaks involving exchangeable protons clearly reveal the stacking of the G:T pairs over the hemi-protonated C:C^+^ base pairs (H1G3(8)-H41/H42/H5C2(7) and H3T1(6)-H6/H5C2(7)), and also the stacking of the T4(9) residues over the G:T base pairs: H1G3(8)-MeT4(9) and H1G3(8)-H4′/H5′/H5″T4(9).

### Solution structure of d<pTCGTTTCGTT>

The three dimensional structure of d<pTCGTTTCGTT> was calculated on the basis of 234 experimental distance constraints by using restrained molecular dynamics methods, and following standard procedures used in our group. Except for the residues T5 and T10, and the corresponding ones in the symmetry related sub-unit, all residues are well defined, with an RMSD of 1.1 Å (Supplementary Table S3). The final AMBER energies and NOE terms are reasonably low in all the structures, with no distance constraint violation >0.5 Å.

**Figure 6.**
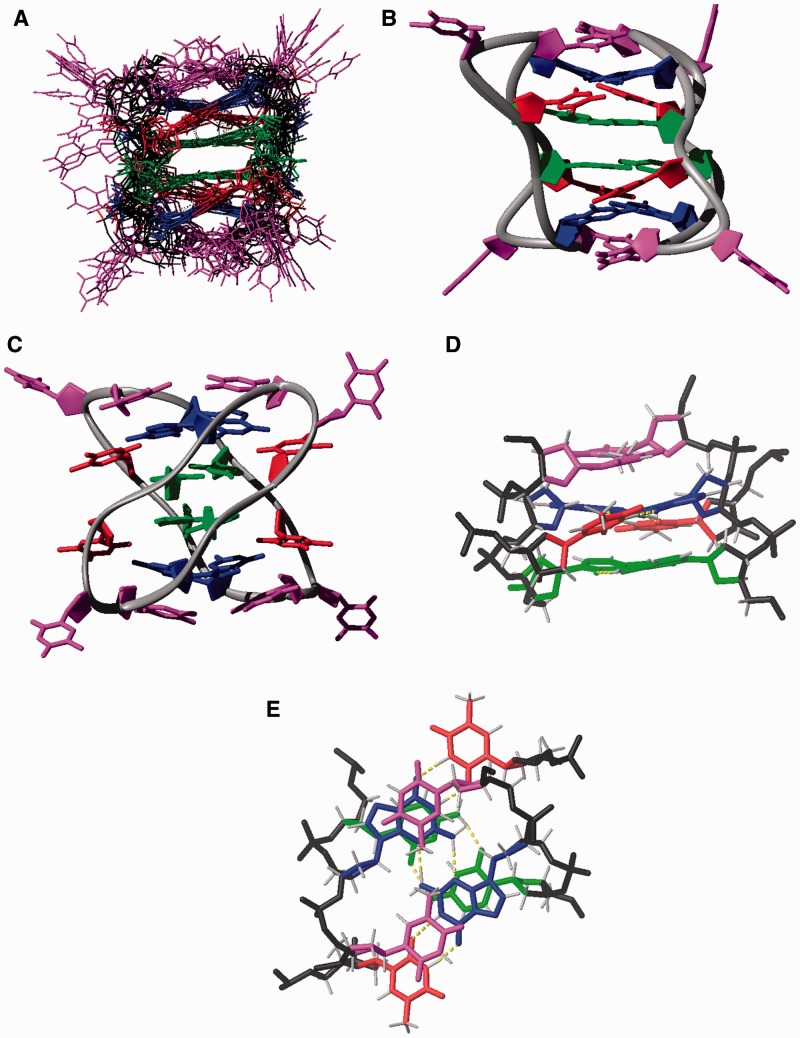
Dimeric structures of d<pTCGTTTCGTT>. Cytosines are shown in green, guanines in blue, thymines involved in GT base pairs in red and unpaired thymines in magenta. Backbone is shown in black. Hydrogen bonds are indicated in yellow. (**A**) Ensemble of the 10 calculated structures. (**B** and **C**) Two views of the overall structure. (**D** and **E**) Details of the stacking interaction between C:C^+^ base pairs, with G:T:G:T minor groove tetrads and capping thymines.

The resulting structure is a dimer consisting of two molecules of d<pTCGTTTCGTT> arranged in an antiparallel way (see schematic representation in Figure 5, left). As reflected by the number of signals in the NMR spectra, the dimer is symmetric. The two decamers associate with each other by forming two intercalated hemi-protonated C:C^+^ base pairs (C2-C12 and C7-C17), sandwiched by four intermolecular G:T base pairs. The base-paired cytidine residues are magnetically equivalent and the characteristic H1′-H1′ contact between their sugars cannot be observed. These two residues stack onto each other through their 5′ side, interacting with the neighboring G:T base pairs through their 3′ side. This kind of interaction explains the lack of amino proton-H2′/H2″ contacts, observed in larger i-motifs in which interaction between C:C^+^ base pairs occurs through their 3′ and 5′ sides alternatively. The G:T base pairs form two G:T:G:T tetrads (G3:T16:G13:T6 and G8:T11:G18:T1) by aligning their minor groove sides. In addition to the four hydrogen bonds of the G:T base pairs, each tetrad is stabilized by two additional intermolecular H-bonds between one of the guanine amino protons and the N3 of the opposite guanine (Figure 5, right). Like other minor groove tetrads (40–43), the two base pairs are not in the same plane (Figure 6), but have a mutual inclination, in this case of around 20°. All glycosidic angles for the guanosines and thymidines involved in the tetrads are *anti*, with values ranging from around −100° for guanosines to −120° to −130° for thymidines. Glycosidic angles for the cytidine residues range from −90° to −110°. All residues adopt predominantly a C2′-endo sugar conformation (*S*-type). Although intercalated cytidine residues in larger i-motif structures usually adopt a C3′-endo sugar conformation, in this case the sugar conformation is probably affected by its proximity to the loop region and the stacked guanine residues.

In addition to the hydrogen bonds, the structure is stabilized by favorable stacking interactions. As can be seen in Figure 6D and E, guanine bases lie just on top of the cytosines. Stacking interactions are also very favorable for the thymines in the first position of the loops (T4 and T9, and the symmetry related ones T14 and T19), which form two caps at both ends of the stacks (Figures 5 and 6). The glycosidic angles of loop residues are also *anti*. These thymines are not base paired, but they interact to each other via a hydrophobic contact between their methyl groups. Several NOEs between the thymine methyl protons with the exchangeable protons of the neighboring base pair provide strong evidence for this orientation (Figure 4). Finally, the thymines in the second position of the loops are disordered.

### Structure of the linear analog d(TCGTTTCGT)

Many of the features of the ^1^H-NMR spectra of d<pTCGTTTCGTT> can be also observed in spectra of the linear sequence d(TCGTTTCGT). Similarities in the NMR spectra of these oligonucleotides are apparent in Figures 3 and 4 and in Supplementary Figure S5. The concentration and pH dependences of the NMR spectra of d(TCGTTTCGT) are also very similar to those of its cyclic analog. However, the number of spin systems observed (14 signals for H6/H8 protons) in the NMR spectra of d(TCGTTTCGT) is not consistent with a single conformation. This fact is especially clear for cytosine residues, where four H5-H6 cross-peaks are observed (Supplementary Figure S9), indicating the presence of two conformations of very similar structure. Although the sequential assignment could not be completed, an important number of cross-peaks could be unambiguously identified (see legends of Supplementary Figures S5 and S7 for a more detailed explanation). Clear sugar-base sequential connectivities H8-H1′/H2′/H2″ are observed between cytosine and guanine residues. Other sequential cross-peaks allow for the identification of thymine H6 protons. Specially relevant are the contacts between guanine H8 and cytosine H6 protons, and a number of cross-peaks involving exchangeable protons of thymines and guanines, indicating the occurrence of G:T wobble base pairs. Also, the minor groove interactions between G:T base pairs is supported by a number of contacts, such as those between H1′ and amino and imino guanine protons (indicated in Figure 5). Stacking of thymines on top of the G:T:G:T tetrads is supported by NOEs between their methyl group and H6/H1′ protons of the adjacent guanine. Since these contacts are observed in the two species, we must conclude that both conformations comprise similar structural elements: two C:C^+^ base pairs, two G:T:G:T tetrads, and stacking of thymine residues on top of them. H1′-H1′ cross-peaks between cytidines through the minor groove could not be observed since their chemical shifts are almost coincident. The lack of amino-H2′/H2″ cross-peaks between stacked cytidines indicate that they stack by its 5′ side as in the case of the cyclic analog. All this structural information is consistent with the co-existence of a head-to-head and a head-to-tail dimerization of d(TCGTTTCGT). Schematic representations of both orientations are given in Supplementary Figure S5. The relative signal intensities indicate an equal population of both species at 5°C, although the head-to-head orientation has a slightly higher thermal stability (Supplementary Figure S9).

### Structural effects of sequence variations: a minor groove G:T:G:T tetrad is necessary to stabilize the mini i-motif

To get further insight into the importance of the interaction between the G:T:G:T tetrad and the C:C^+^ base pair in the stability of these structures, we have explored the structure of the cyclic decamer d<pTGCTTTGCTT>, which contains the same nucleotides in a slightly different order (CG to GC permutations), as well as the linear octamer d(TCGTTCGT), in which the length of the loop has been reduced in 1 nt (Figure 1).

#### Structure of d<pTGCTTTGCTT>

As can be observed in Supplementary Figure S10, the NMR spectra of d<pTGCTTTGCTT> exhibit very different features compared to those of d<pTCGTTTCGTT>. No imino signals are observed around 15 ppm. Instead, a sharp imino signal is observed at 13.5 ppm, characteristic of G:C base pairs. Analysis of two-dimensional spectra indicates that d<pTGCTTTGCTT> forms a monomeric dumbbell-like structure (data not shown). Most interestingly, the same spectral features are found at acidic pH (Supplementary Figure S10), indicating that CG to GC permutations in the sequence destabilizes the i-motif completely.

#### Structure of d(TCGTTCGT)

In the case of the linear octamer d(TCGTTCGT), NMR spectra indicate the formation of hemi-protonated C:C^+^ base pairs (Figure 3C and Supplementary Figure S6). As in d(TCGTTTCGT), the concentration and pH dependence of the NMR spectra indicates the formation of a dimeric structure. Contrary to the case of d(TCGTTTCGT), only eight spin system are detected, indicating the formation of a single conformation with a 2-fold symmetry. Sequential connectivities were observed in the NOESY experiments for the residues T1→C2→G3→T4 and T5→C6→G7 (Supplementary Figure S7, left). Two imino proton signals appeared in the characteristic region of hemi-protonated C:C^+^ base pairs. Other two imino proton signals were observed at 10.11 ppm and 11.36 ppm (T1 and G7, respectively) that showed an intense cross-peak between them indicating the formation of a mismatched G:T base pair. The amino protons of G7 are not hydrogen bonded and exchange rapidly with the solvent. Another imino proton signal was observed at 10.35 ppm (G3). Although it is not hydrogen bonded, it showed cross-peaks with 2 amino protons at 8.64 ppm and 6.00 ppm. The amino proton at 8.64 ppm showed a cross-peak with an H1′ proton at 5.80 ppm. These connectivities are consistent with the formation of a G:G base pair between G3 and the symmetry related guanine (G11). Other structurally important cross-peaks revealed stacking interactions between residues: H3T1(9)-H5C2(10), H1G7(15)-H41C2(10), H1G3(11)-H41C6(14) and MeT4(12)- H8/H1′/H2′H2″G3(11).

Overall, we can conclude that d(TCGTTCGT) adopts a head-to-head dimeric structure, similar to that adopted by d(TCGTTTCGT) (see Supplementary Figure S6 for schematic representation of the two possible arrangements). In this case, the two hemi-protonated C:C^+^ base pairs, C6-C14 and C2-C10, are surrounded, in one side, by two G:T base pairs, most probably forming a G:T:G:T tetrad (involving G7 and T1 and their symmetry related residues G15 and T9), and in the loop side by a G:G base pair formed by G3 and G11. Interestingly, the presence of only two thymines in the loop region seems to prevent the formation of the minor groove tetrad on this side of the structure. The head-to-tail orientation is not supported by the NMR data since such arrangement requires the formation of hemi-protonated C:C^+^ base pairs between non-equivalent cytidine residues, and a minor groove tetrad involving residues G7:T13:G11:T1.

#### Thermal stability

CD and NMR-monitored thermal denaturation experiments were carried out to analyse the relative stabilities of d<pTCGTTTCGTT>, d(TCGTTTCGT) and d(TCGTTCGT) (Figure 2). CD melting curves were fitted to a two-state homodimerization process (65) to obtain the thermodynamic parameters summarized in Table 1. This approximation is not strictly valid in the case of d(TCGTTTCGT), where two dimeric structures co-exist in equilibrium. Although the relative intensity of cytosine H5-H6 cross-peaks in the TOCSY experiments indicates that the head-to-head orientation is slightly more stable than the head-to-tail one (Supplementary Figure S9), the difference is not enough to observe two transitions in the CD melting curves and, therefore, the thermal denaturation process can be described by a single apparent melting temperature. Melting experiments for d(TCGTTCGT) were carried out at a higher oligonucleotide concentration and in the presence of 10 mM Mg^2+^, since the melting temperature in the same salt conditions as the other two sequences was too low to extract reliable thermodynamic parameters. Comparison between the apparent free energies of d(TCGTTTCGT) and d(TCGTTCGT) clearly shows the additional stability conferred by the presence of two G:T:G:T tetrads in the structure instead of only one.

**Table 1.** Thermodynamic parameters for the dimerization process at pH 4.5

Sequence	*T*_m_	Δ*G*°_298_	Δ*H*°	*TΔS*°_298_
d<pTCGTTTCGTT>^a^	42.0 ± 0.5	−7.9 ± 0.3	−38.1 ± 0.2	−30.2 ± 0.2
d(TCGTTTCGT)^a^^,^^c^	30.6 ± 0.2	−6.3 ± 0.6	−44.7 ± 0.3	−38.4 ± 0.3
d(TCGTTCGT)^b^	28.4 ± 0.5	−4.8 ± 1.8	−42.8 ± 0.9	−38.0 ± 2.8

Values of *T*_m_ are expressed in °C and Δ*G*°_,_ Δ*H*° and *TΔS*° in kcal·mol^−1^.

^a^*Experimental conditions*: 20 mM sodium acetate, 200 mM NaCl. [d<pTCGTTTCGTT>] = 50 µM, [d(TCGTTTCGT)] = 100 µM.

^b^*Experimental conditions*: 20 mM sodium acetate, 100 mM NaCl, 10 mM MgCl_2_. [d(TCGTTCGT)] = 0.66 mM.

^c^Apparent thermodynamic parameters for a single denaturation process.

### Competition with duplex structures

NMR experiments of the equimolar mixture of d(TCGTTTCGT) and its complementary strand d(ACGAAACGA) were carried out under different pH conditions. As expected, the duplex structure is predominant at neutral pH. The duplex is also more stable at pH 5. However, the mini i-motif structure is the major species at pH 4, as can be observed in Supplementary Figure S11. No differences were observed upon sample preparation under different annealing procedures, indicating that both i-loop and duplex formation does not exhibit a very different kinetic behavior. It is worth mentioning that at pH 4 the imino region of the NMR spectra of d(ACGAAACGA) indicates the formation of a non-canonical structure, which is not monomeric according to native gel electrophoresis experiments (Supplementary Figure S4). A more detailed study of this structure is in progress and will be reported in due time.

## DISCUSSION

The most remarkable feature of the dimeric structure of d<pTCGTTTCGTT> and its linear analogs d(TCGTTTCGT) and d(TCGTTCGT) is that oligonucleotides with no contiguous cytosine residues in their sequence can fold into an i-motif structure. To our knowledge, this is the first time that a minimal i-motif with only two C:C^+^ pairs has been observed. The study of this unusual structure has been facilitated by the use of a cyclic oligonucleotide, which provides a very convenient way to simplify the structural problem. Whereas the NMR spectra of d(TCGTTTCGT) is complicated by the co-existence of two very similar species, arising from head-to-tail and head-to-head association, in the cyclic analog the two dimerization modes give rise to a single structure. The conformational restriction imposed by the cyclization of the sugar-phosphate backbone not only diminishes the number of possible competing structures, but also enforces a pre-organization that decreases the associated entropic penalty. The result is that this pre-organization can facilitate intermolecular interactions that are difficult to observe in linear oligonucleotides. However, we must underline that cyclization is not a prerequisite for the formation of this mini i-motif, since the linear oligonucleotides d(TCGTTTCGT) and d(TCGTTCGT) adopt very similar structures as their cyclic analog. In fact, thermodynamics parameters shown in Table 1 clearly indicate that the enhanced stability of d<pTCGTTTCGTT> with respect to its linear analog is mainly entropic in nature.

The stability of i-motif structures greatly depends on the number of hemi-protonated C:C^+^ base pairs. Thus, hairpin dimerization through C:C^+^ base pairs occurs in the insulin mini-satellite sequence d(CCCCTGTCCC), which dimerizes forming a stem of eight C:C^+^ base pairs (66). In the 11-nucleotide fragment of the CENP-B box d(TCCCGTTTCCA), the presence of three consecutive cytosine residues allows the formation of a stem of five hemi-protonated C:C^+^ base pairs (23). Moreover, the different dimers formed by variations of the sequence d(CCTTTACC) (67) were probably the smallest i-motif structures known prior to the present work.

It is important to notice that the mini i-motifs described here retain a significant stability at neutral pH. The particular pH value of the midpoint transition is an appropriate indicator of the stability of i-motif structures. The apparent p*K*_a_ value of 5.9 obtained for the midpoint transition of d<pTCGTTTCGTT> was comparable to the values obtained for the sequences derived from the CENP-B box (68) and it agrees with those obtained for pure i-motif structures with small connecting loops (2-4 residues) (69). Also, the *T*_m_ and the calculated value of Δ*G* = −7.9 kcal·mol^−^^1^ for the formation of the dimeric structure of d<pTCGTTTCGTT> (Table 1) is not too different to those obtained for other dimeric i-motif structures with longer C:C^+^ tracks, such as that of d(TCCCGTTTCCA) at pH 4.6 with an associated Δ*G* = −8.5 kcal·mol^−^^1^ (68). These results reveal that formation of only two hemi-protonated C:C^+^ base pairs can lead to an apparent p*K*_a_ comparable to those of cytosine rich sequences.

Although the number of hemi-protonated base pairs is important for stability, capping interactions are also determinant for i-motif formation at slightly acidic or neutral pH. In the dimeric structures of d(TCGTTTCGT) and its cyclic analog d<pTCGTTTCGTT>, an important factor in the stabilization of the mini i-motif is the neighboring G:T:G:T minor groove tetrads that surround the hemi-protonated C:C^+^ base pairs, as well as other stabilizing capping interactions in the loops. This effect becomes more evident when comparing the structures of the linear oligomers d(TCGTTTCGT) and d(TCGTTCGT). The lower stability of d(TCGTTCGT) is most probably due to the conformational restriction imposed by a shorter loop, that presumably prevents the formation of a full tetrad in that side of the structure. Thus, the mini i-motif formed in the dimeric structure of d(TCGTTCGT) is stabilized by only one minor groove tetrad at the open side of the structure. In contrast, the two structures of d(TCGTTTCGT) exhibit an enhanced stability, since the i-motif is sandwiched by minor groove tetrads at its both sides. The importance of the interaction between C:C^+^ base pairs and G:T:G:T tetrads becomes apparent in the case of d<pTGCTTTGCTT>, where the lack of the G:T:G:T tetrads destabilizes the i-motif completely even at acidic pH. All this evidence indicates that minor groove tetrads play an essential role for the stabilization of these i-motif structures. The minor groove G:T:G:T tetrad found in these structures has been also found in the solution structure of the cyclic oligonucleotide d<pTCGTATGT> (45). Presumably, other similar minor groove tetrads reported in the bibliography, as those formed by G:C:G:C or A:T:A:T (41,42), would be also able to stabilize hemi-protonated C:C^+^ base pairs. This kind of favorable interaction between two different structural elements is one of the few cases where two DNA structural motifs have been found within a single oligodeoxynucleotide. Interestingly, this is not the only case, since Nonin-Lecomte *et al.* have found that a 24-mer containing the G- and C-rich stretches from the human mitochondrial DNA self-associates into a mixed triplex/G-tetrad structure (70).

Since the GTTT sequence involved in the formation of the minor groove G:T:G:T tetrads corresponds to a highly conserved region of the CENP-B box (48,49), it is tempting to consider that these minor groove tetrads could have been evolutionarily selected to stabilize the centromeric i-motif structures that would eventually confer the centromere geometry and architectural constraints essential for the fidelity of chromosome segregation (71). The bidirectional transcription of centromeric DNA repeats ensures the accessibility of the DNA strands (72,73), allowing the hypothetical formation of intercalary motifs. The recent detection of functional centromeric polymorphisms at an endogenous human centromere has revealed that centromere specification requires the cooperation of structural and epigenetic mechanisms (74). The study shows that human alpha-satellite arrays with more CENP-B boxes seem to be more suitable for centromere assembly. The above scenario is in agreement with the hypothesis that a specific sequence-independent structural motif is also required for centromere identity (75,76). In line with this suggestion, the unexpected observation of i-motif like structures in sequences with low cytosine content reduces the sequence requirements for i-motif formation. Therefore, i-motif like structures might be more common in chromosomes than previously anticipated. Here, it is important to highlight that oligonucleotides forming mini i-motifs may remain unnoticed when analysing their structure by CD spectroscopy. CD profiles obtained for these mini i-motifs do not resemble the typical CD spectra of i-motifs (62) but are more similar to CD spectra of structures stabilized by minor groove tetrads (41,45). The slipped G:T:G:T tetrads present in these structures have a larger effect on the CD spectra than the C:C^+^ intercalated base pairs. This effect should be considered when analysing the CD spectra of oligonucleotides susceptible of forming i-motifs.

Finally, the formation of i-motifs in DNA fragments with very few cytosines implies that their complementary strands are not guanine-rich and, consequently, the competing duplex is less stable, as it has a low GC content. Also, the isolated complementary strand does not have a tendency to form G-quadruplex. We have observed that d(TCGTTTCGT) can form a mini i-motif at acidic pH even in the presence of the complementary strand and, most importantly, its complementary strand also adopts an alternative non-canonical structure at acidic pH, different than the G-quadruplex. The co-existence of i-motif, duplex and this unknown structure may have interesting biological implications and clearly deserves further investigations.

## CONCLUSION

Our discovery of minimal i-motifs stabilized by minor groove tetrads, both in cyclic oligonucleotides and in biologically relevant linear sequences, is a singular example of the ability of DNA to integrate different structural elements to furnish tertiary DNA structures. Moreover, the described structures have also shown that i-motif formation is not only limited to cytosine rich sequences in acidic conditions. These findings rekindle the interest of i-motif’s physiological importance and biotech applications.

## ACCESSION NUMBERS

Atomic coordinates of d<pTCGTTTCGTT> have been deposited in the Protein Data Bank (accession number 2LSX).

## SUPPLEMENTARY DATA

Supplementary Data are available at NAR Online: Supplementary Tables 1–3 and Supplementary Figures 1–11.

## FUNDING

Spanish MICINN [CTQ2010-21567-C02-01/02 and CSD2009-80]; Generalitat de Catalunya [2009SGR-208 and Xarxa de Referència en Biotecnologia]; MINECO [BFU2011-30295-C02-01 to A.V.]; an institutional grant from Fundación Ramón Areces to the CBMSO. Funding for open access charge: MICINN.

*Conflict of interest statement*. None declared.

## Supplementary Material

Supplementary Data
