# Supplementary material for: A minimal i-motif stabilized by minor groove G:T:G:T tetrads
Source: Nucleic Acids Res. 2012 Oct 5;40(22):11737–47. doi: 10.1093/nar/gks911 (PMC3526289; doi:10.1093/nar/gks911)
Supplement: Supplementary Data [file supp_40_22_11737__index.html]

A minimal i-motif stabilized by minor groove G:T:G:T tetrads — A minimal i-motif stabilized by minor groove G:T:G:T tetrads — Supplementary Data 

# A minimal i-motif stabilized by minor groove G:T:G:T tetrads

## Supplementary Data

files

**Files in this Data Supplement:**

- Supplementary Data - pdf file
