# Supplementary material for: A minimal i-motif stabilized by minor groove G:T:G:T tetrads
Source: Nucleic Acids Res. 2012 Oct 5;40(22):11737–47. doi: 10.1093/nar/gks911 (PMC3526289; doi:10.1093/nar/gks911)
Supplement: Supplementary Data [file supp_gks911_nar-01351-f-2012-File008.pdf]

Suplemmentary DATA FOR:

A minimal i-motif stabilized by minor groove G:T:G:T tetrads

Núria Escaja[2], Júlia Viladoms[2], Miguel Garavís[1,3], Alfredo Villasante[3], Enrique Pedroso[2,]* and Carlos González[1,]*

Tables and Figures mentioned in the main text:

Table S1. Assignment list of d<pTCGTTTCGTT> at pH 5.1, T=5ºC (25 mM phosphate buffer, 100 mM NaCl)
Table S2: Assignment list of d(TCGTTCGT) at pH 5, T=5ºC (25 mM phosphate buffer, 100 mM NaCl, 15 mM $MgCl_2$)
Table S3. Experimental constraints and calculation statistics for d<pTCGTTTCGTT>.

Figure S1. Schematic representation of G-tetrad (A), hemiprotonated C:C^+ base pair (B), and major and minor groove G:T:G:T tetrads (C and D, respectively).
Figure S2. NMR spectra of d<pTCGTTTCGTT> in H_2O/D_2O 9:1 at T = 5 ºC in 25 mM phosphate buffer, pH 7, 100mM NaCl. Top: low concentration (80 μM); Bottom: high concentration (800 μM).
Figure S3. Top: NMR spectra of d<pTCGTTTCGTT> at different temperatures and 80 μM oligonucleotide concentration. Bottom: NMR spectra of d(TCGTTTCGT) at different temperatures and 0.5 mM, 0.1 mM and 0.01 mM oligonucleotide concentration. All spectra are in H_2O/D_2O 9:1, 25 mM phosphate buffer, 100 mM NaCl, pH 5.0.
Figure S4: Non denaturing gel electrophoresis. (A) Non denaturing 20% PAGE in TBE buffer pH 8.3, 25 mM NaPi and 100 mM NaCl. (B) Non denaturing 20% PAGE in 10 mM Robinson-Britton buffer pH 4, 100 mM NaCl. Lanes: (**1**) dT ladders, (**2**) d(TCGTTTCGT), (**3**) d(AGCAAAGCA), (**4**) 1:1 mix of d(TCGTTTCGT) and d(AGCAAAGCA), (**5**) d(TCCGTTTCCGT), (**6**) TBA(Thrombin binding aptamer) d(GGTTGGTGTGGTTGG).
Figure S5. Exchangeable protons region of the NOESY spectrum (t_m=250 ms) of d(TCGTTTCGT) in H_2O/D_2O 9:1 in 25 mM phosphate buffer, pH 5, T=5ºC, 100 mM NaCl, 0.5 mM oligonucleotide concentration and schematic representations of proposed head-to-head and head-to-tail dimeric structures.
Figure S6. Exchangeable protons region of the NOESY spectrum (t_m=200 ms) of d(TCGTTCGT) in H_2O/D_2O 9:1 in 25 mM phosphate buffer, pH 5, T=5ºC, 100 mM NaCl, 10 mM $MgCl_2$, 0.66 mM oligonucleotide concentration, and a schematic representation of the proposed head-to-head dimeric structure**.**
Figure S7. Non-exchangeable protons region of the NOESY spectra of d(TCGTTCGT) (t_m=200 ms) (Left) and d(TCGTTTCGT) (t_m=250 ms) (Right) in H_2O/D_2O 9:1 in 25 mM phosphate buffer, pH 5, T=5ºC, 100 mM NaCl. Same oligonucleotide concentrations as in S5 and S6.
Figure S8. Proton connectivity map of d<pTCGTTTCGTT>.
Figure S9. Cytosine H6-H5 cross-peaks region of the TOCSY spectra of d(TCGTTTCGT) at different temperatures (25 mM phosphate buffer, pH 5, 100 mM NaCl, 0.5 oligonucleotide concentration).
Figure S10. NMR spectra of d<pTGCTTTGCTT> in H_2O/D_2O 9:1 in 25 mM phosphate buffer, 100 mM NaCl T= 5ºC. Top) pH 7.0 Bottom) pH 4.0, 0.5 mM oligonucleotide concentration.
Figure S11. Duplex competition experiments. NMR spectra of: (A) d(TCGTTTCGT) at pH 4.5, T=5ºC (100 μM oligonucleotide concentration, 25 mM phosphate buffer, 100 mM NaCl); (B) Complementary strand d(AGCAAAGCA) at pH 4.5, T=5ºC (100 μM oligonucleotide concentration, 25 mM phosphate buffer, 100 mM NaCl); (C, D and E) Equimolar mixture of d(TCGTTTCGT) and d(AGCAAAGCA), T=5ºC, 100 μM oligonucleotide concentration, 25 mM phosphate buffer, 100 mM NaCl at pH 7, 5 and 4.5, respectively.

SUPPLEMENTARY TABLES

Table S1. Assignment list of d<pTCGTTTCGTT> at pH 5.1, T=5ºC

Buffer conditions: 25 mM phosphate buffer, 100 mM NaCl

	H1/H3	H42/H22	H41/H21	H6/H8	H5/Me	H1’	H2’	H2”	H3’	H4’	H5’	H5”
T1	11.85	-	-	7.85	1.99	6.49	2.55		4.97	4.38	4.12	
C2	15.42	9.52	7.58	7.49	6.31	6.35	1.06	2.15	4.82	4.56	4.10	
G3	10.85	8.83	5.69	8.38	-	5.95	3.00	2.68	5.15	4.41	3.9, 4.11	
T4	11.5	-	-	7.63	1.76	6.08	2.07	2.33	4.81	3.99		
T5	10.47	-	-	7.77	1.92	6.43	2.28	2.55	4.63	4.47	3.98, 4.13	

Table S2. Assignment list of d(TCGTTCGT) at pH 5, T=5ºC

Buffer conditions: 25 mM phosphate buffer, 100 mM NaCl, 10 mM $MgCl_2$*

	H1/H3	H42/H22	H41/H21	H6/H8	H5/Me	H1’	H2’	H2”	H3’	H4’	H5’	H5”
T1	11.36	-	-	7.52	1.79	6.25	2.56	2.39	4.83	n.a.	n.a.	
C2	15.40	9.40	7.44	7.26	6.42	6.23	0.98	2.21	4.70	3.73	n.a.	
G3	10.36	8.64	6.00	8.14	-	5.80	2.76	2.51	4.95	n.a.	n.a.	
T4	**	-	-	7.34	1.51	5.72	1.85	2.02	4.27	n.a.	n.a.	
T5	**	-	-	7.61	1.74	6.11	2.40	2.52	n.a.	n.a.	n.a.	
C6	15.34	9.36	7.42	7.30	6.24	6.21	0.95	2.27	4.68	4.46	n.a.	
G7	10.10	n.o.		8.20	-	5.69	2.78	2.50	4.96	n.a.	n.a.	
T8	**	-	-	7.42	1.56	6.14	2.41, 1.97		n.a.	n.a.	n.a.	

*10 mM of $MgCl_2$ was added to the sample to obtain a higher thermal stability. Proton chemical shifts at T=5ºC do not differ from those obtained for the dimeric structure when only Na^+ was added.

**Chemical shifts of imino protons of T4, T5 and T8 are: 10.44, 10,71 and 11.21 ppm, but it was not possible to assign them.

n.o: not observed

n.a: not assigned

**Table S3**: Experimental constraints and calculation statistics of d<pTCGTTTCGTT>		
Experimental distance constraints		
Total number	234	
intra-residue	112	
sequential	70	
range > 1	52	
Intra-subunit	190	
Inter-subunit	44	
RMSD (Å)		
all well-defined bases[+]	1.1±0.3 Å	
all well-defined heavy atoms[+]	1.6±0.4 Å	
backbone	2.1±0.5 Å	
all heavy atoms	2.7±0.6 Å	
Residual violations	Average	Range
Sum of violation (Å)	3.4	2.4 .. 3.8
Max. violation (Å)	0.36	0.21 .. 0.47
NOE energy (kcal/mol)	12	8 .. 13
Total energy (kcal/mol)	-13500	-1219.. -2460

[+]All except unpaired thymines (4, 5, 9 and10).

SUPLEMENTARY FIGURES

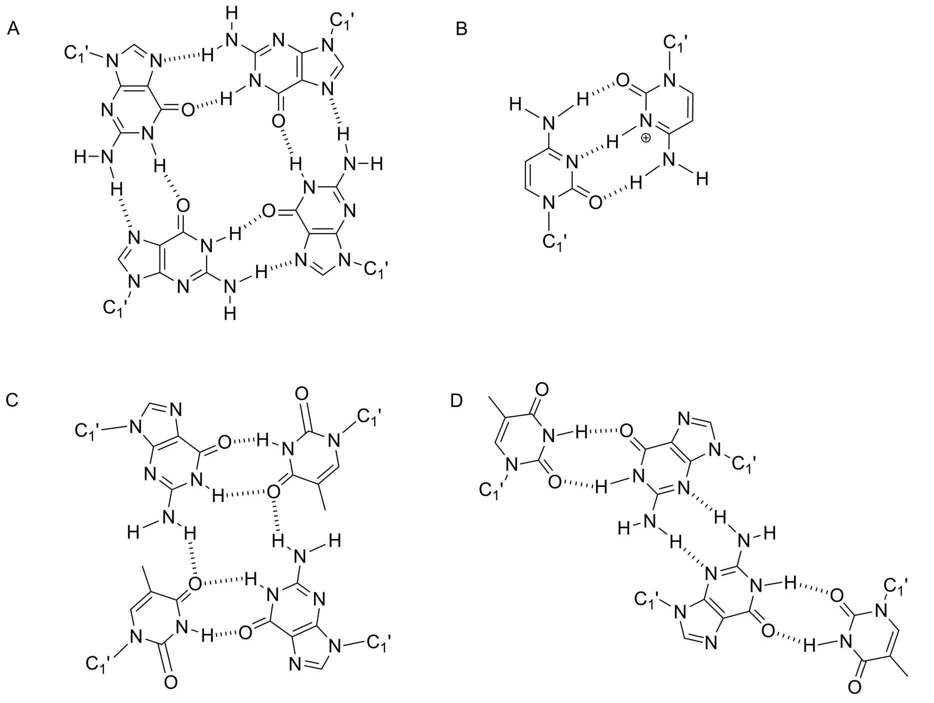

Figure S1. Schematic representation of G-tetrad (A), hemiprotonated C:C$^+$ base pair (B) and major and minor groove G:T:G:T tetrads (C and D, respectively).

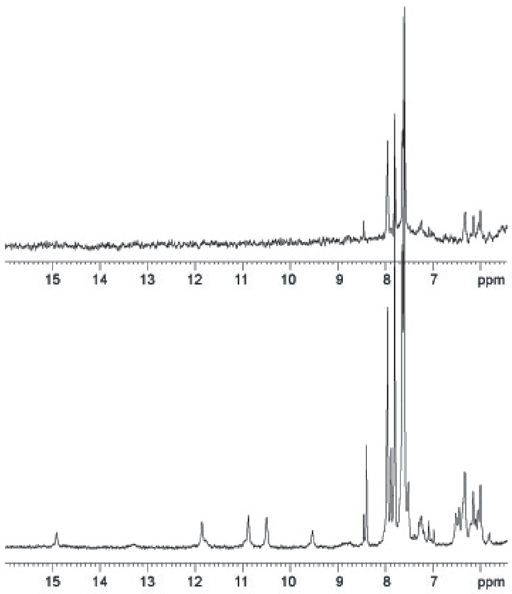

Figure S2. NMR spectra of d<pTCGTTTCGTT> in H_2O/D_2O 9:1 at T = 5 ºC in 25 mM phosphate buffer, pH 7, 100mM NaCl. Top: low concentration (80 μM); Bottom: high concentration (800 μM).

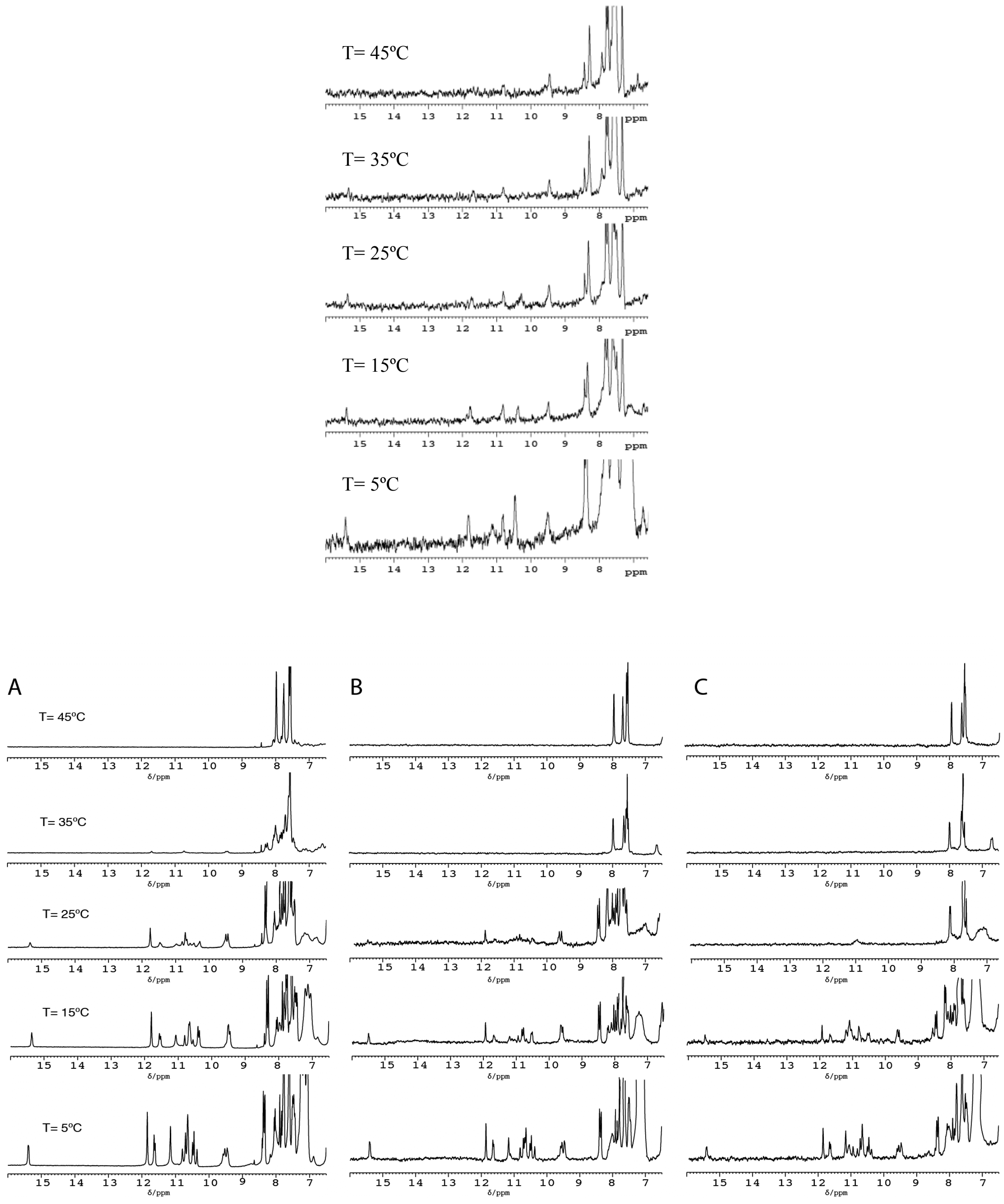

Figure S3. Top: NMR spectra of d<pTCGTTTCGTT> at different temperatures and 80 μM oligonucleotide concentration. Bottom: NMR spectra of d(TCGTTTCGT) at different temperatures and 0.5, 0.1 and 0.01 mM oligonucleotide concentration (A, B and C, respectively). All spectra are in H_2O/D_2O 9:1, 25 mM phosphate buffer, 100 mM NaCl, pH 5.0.

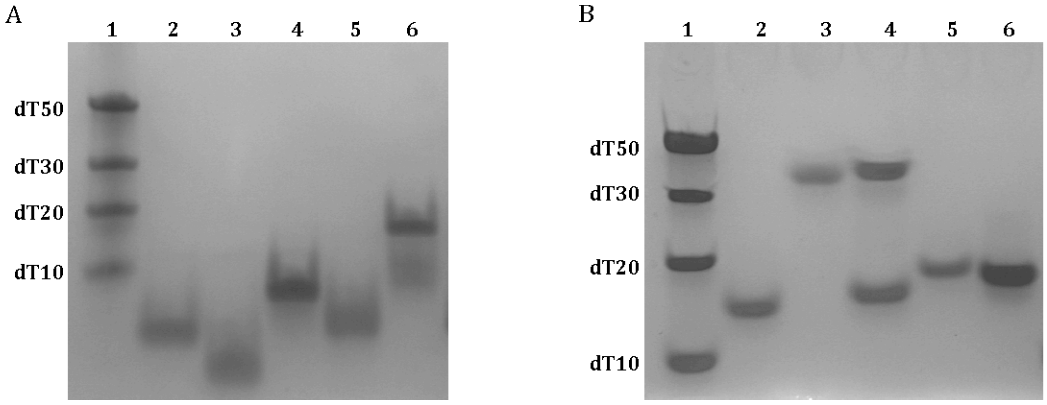

Figure S4. Non denaturing electrophoresis. (A) Non denaturing 20% PAGE in TBE buffer pH 8.3, 25 mM NaPi and 100 mM NaCl. (B) Non denaturing 20% PAGE in 10mM Robinson-Britton buffer pH 4, 100 mM NaCl. (**2**): d(TCGTTTCGT), (**3**): d(AGCAAAGCA), (**4**): 1:1 mix of d(TCGTTTCGT) and d(AGCAAAGCA), (**5**): d(TCCGTTTCCGT), (**5**): TBA (Thrombin binding aptamer) d(GGTTGGTGTGGTTGG). Lane (**1**): dT ladders.

Experimental methods on gel electrophoresis

Electrophoresis experiments were performed with 10 x 7cm native gel containing 20% polycrylamide (Acrylamide:Bis-acrylamide 19:1 ratio) in TBE Buffer pH 8.3 supplemented with 25 mM NaPi and 100 mM NaCl (Figure S4A) or in Robinson-Britton Buffer ([CH_3COOH]= [H_3PO_4] = [H_3BO_3] =10 mM) pH 4 supplemented with 100 mM NaCl (Figure S4B). The samples were prepared at 80 μM concentration and were incubated overnight at 4ºC in TBE Buffer pH 8.3, 25 mM NaPi and 100 mM NaCl (Figure S4A) or in 40 mM Robinson-Britton Buffer pH 4 and 100 mM NaCl (Figure S4B). Gels were loaded after 1h prerunning time and electrophoresis was performed at 10V/cm (Figure 4SA) or 8.5V/cm (Figure 4SB) for 90 and 180 min, respectively, at 4ºC. Gels were viewed after staining with Stains-All dye (Sigma E-9379).

Results

d(TCGTTTCGT) (lane 2) exhibits a larger mobility than TBA (a monomeric 15mer quadruplex) (lane 6) at pH 8.3, but their mobility is more similar at pH 4. This is consistent with monomeric and dimeric species of d(TCGTTTCGT) at pH 8.3 ant 4, respectively. d(TCCGTTTCCGT) (lane 5) is a variation of d(TCGTTTCGT) with two additional cytosines and exhibits a similar behaviour. This oligonucleotide most probably forms a dimeric i-motif with four hemiprotonated $C:C^+$, and it has been included in this experiment as an additional control.

d(AGCAAAGCA) (lane 3) presents very different mobility at different pHs. At neutral pH is monomeric and most probably unstructured, but it forms some higher order structure at acidic pH (maybe tetrameric).

The equimolar mix of d(TCGTTTCGT) and d(AGCAAAGCA) (lane 4) exhibits a single spot at pH 8 (a single duplex structure). However, two species are clearly observed at pH 4, consistent with the dimeric i-motif described in this manuscript, and a higher order structure adopted by d(AGCAAAGCA).

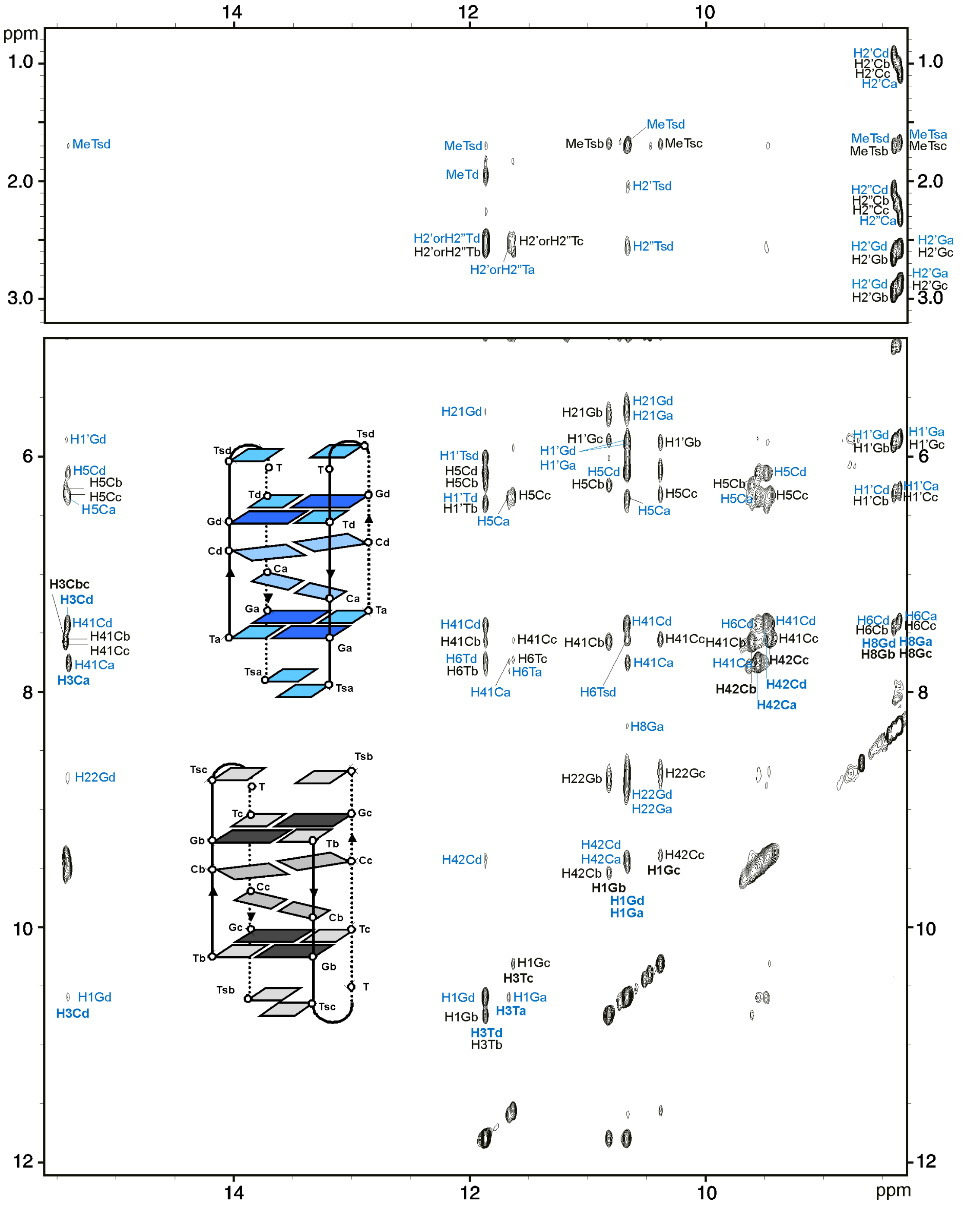

Figure S5. Exchangeable protons region of the NOESY spectrum (t_m = 300 ms) of d(TCGTTTCGT) in H_2O/D_2O 9:1 in 25 mM phosphate buffer, pH 5, T=5ºC, 100 mM NaCl, 0.5 mM oligonucleotide concentration and schematic representations of proposed head-to-head and head-to-tail dimeric structures. Signals corresponding to the head to head orientation are labelled in blue and the signals corresponding to the head to tail dimer are labeled in black.

Thymine residues are highly overlapped as only six Me-H6 cross-peaks can be distinguished in the TOCSY spectra (not shown). Only those thymine residues that show stacking interactions or are involved in the G:T:G:T tetrads can be identified and labeled according to their contacts with guanine and cytosine residues. Four spin systems corresponding to cytosines are clearly observed, both in the exchangeable and non exchangeable proton region (Figures S7 and S9). These residues exhibit imino-amino proton cross-peaks in the characteristic region of hemiprotonated $C:C^+$ base pairs (labeled Ca,Cb,Cc and Cd in the Figure). Two of these $C:C^+$ base pairs are formed by equivalent cytosines (Ca and Cd, head to head orientation) and one of them is formed by two different cytosines (Cb and Cc, head to tail orientation). Four spin systems correspond to guanines and show cross-peaks that reveal their implication in minor groove G:T:G:T tetrads (H1-H3 imino-imino proton cross-peaks between guanine and thymine residues and guanine amino-H1' contacts through the minor groove). Based on guanine amino-H1' cross-peaks, two of the G:T:G:T tetrads are formed by two equivalent guanines (head to head orientation) and one of the tetrads is formed by two non equivalent guanines (head to tail orientation).

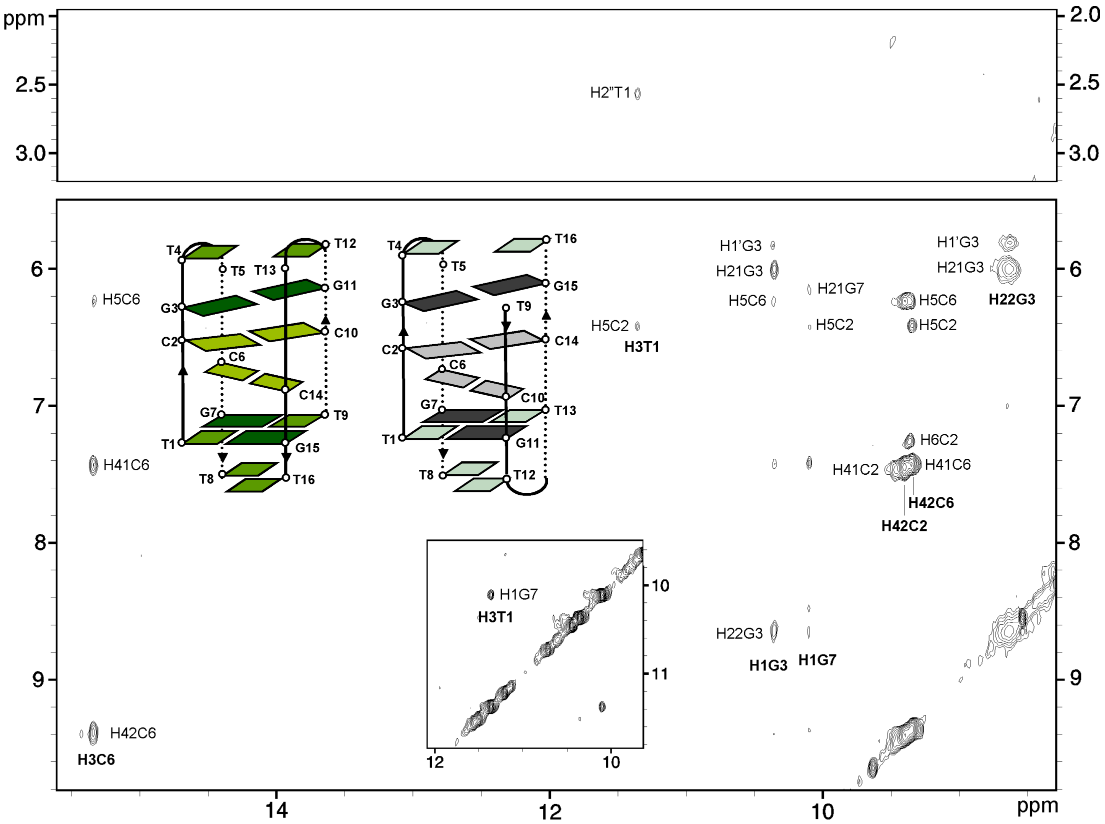

Figure S6. Exchangeable protons region of the NOESY spectrum (t_m = 200 ms) of d(TCGTTCGT) in H_2O/D_2O 9:1 in 25 mM phosphate buffer, pH 5, T=5ºC, 100 mM NaCl, 15 mM $MgCl_2$, 0.66 mM oligonucleotide concentration, and schematic representation of the proposed head-to-head dimeric structure (green coloured) and the alternative not observed head-to-tail structure. According to the observed H3-H41/H42/H5 crosspeaks, the most stable hemiprotonated $C:C^+$ base pair is the one formed between equivalent cytosine residues. This rules out the head-to-tail orientation, since the H3 proton of protonated cytosine would show cross-peaks with the amino protons of a non equivalent base-paired cytosine (C6 and C10).

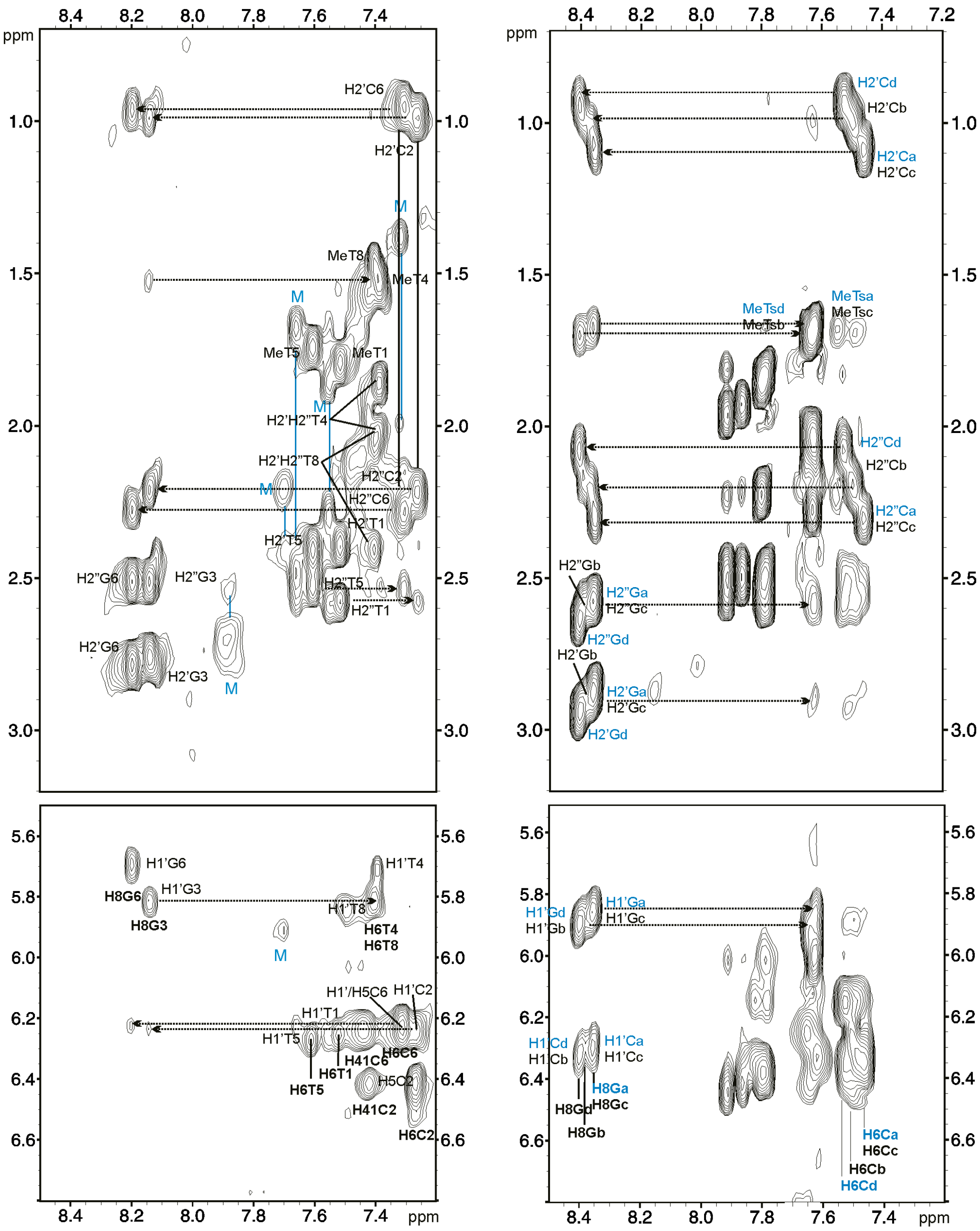

Figure S7. Non-exchangeable protons region of the NOESY spectra of d(TCGTTCGT) (t_m = 200 ms) (Left) and d(TCGTTTCGT) (t_m = 250 ms) (Right) in H_2O/D_2O 9:1 in 25 mM phosphate buffer, pH 5, T=5ºC, 100 mM NaCl. Signals corresponding to the unstructured species of d(TCGTTCGT) have been labelled with an M (left panel). In the right panel, signals corresponding to the head-to-head and the head-to-tail dimeric structures of d(TCGTTTCGT) are labelled in blue and black, respectively. Oligonucleotide concentrations are the same as in S5 and S6.

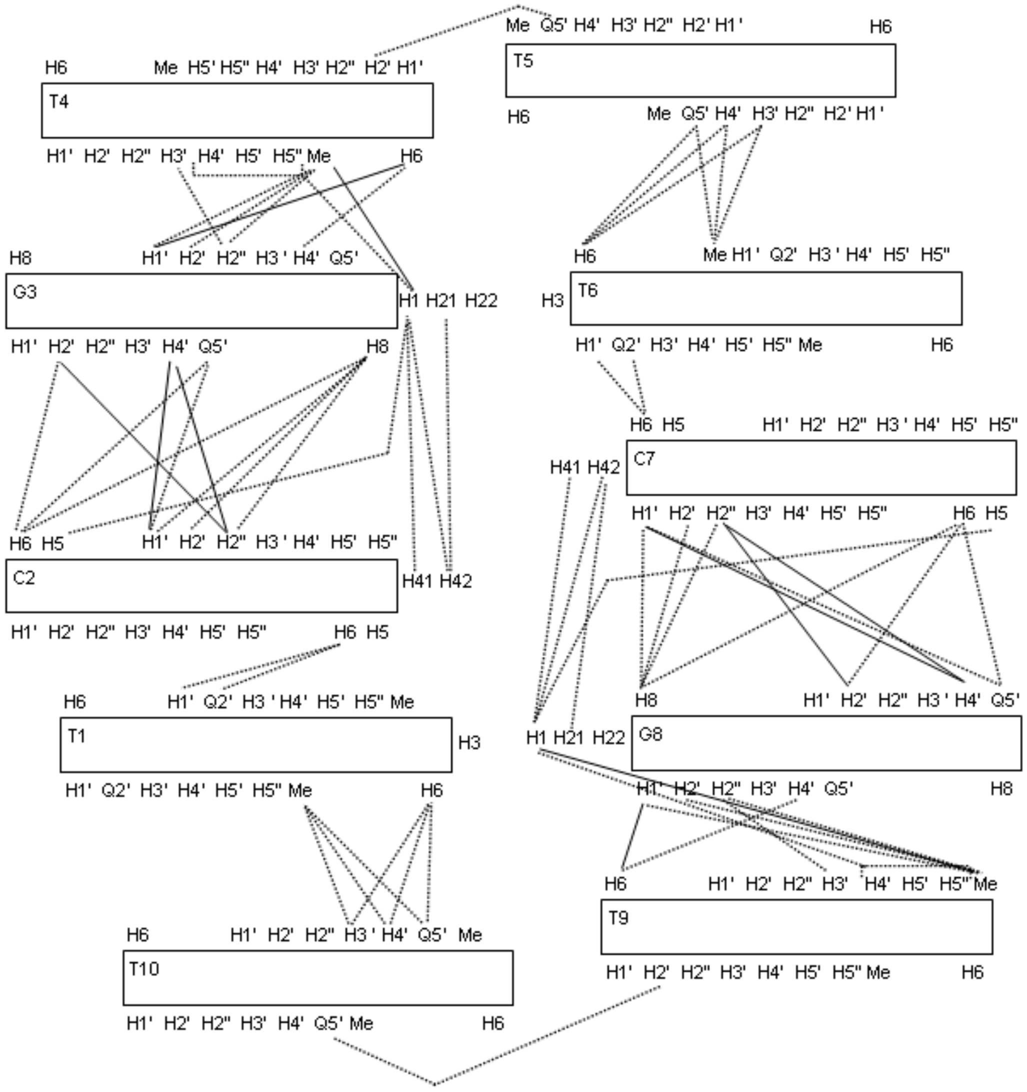

Figure S8. Proton connectivity map of d<pTCGTTTCGTT>.

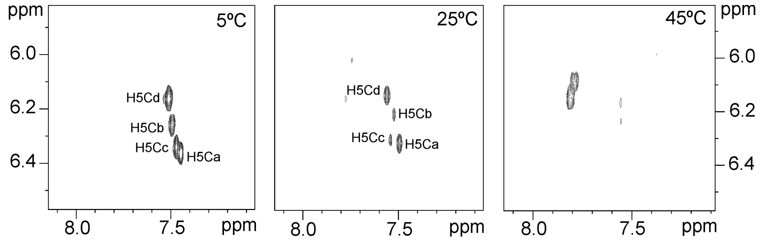

Figure S9. Cytidine H6-H5 cross-peaks region of the TOCSY spectra of d(TCGTTTCGT) at different temperatures (25 mM phosphate buffer, pH 5, 100 mM NaCl, 0.5 oligonucleotide concentration).

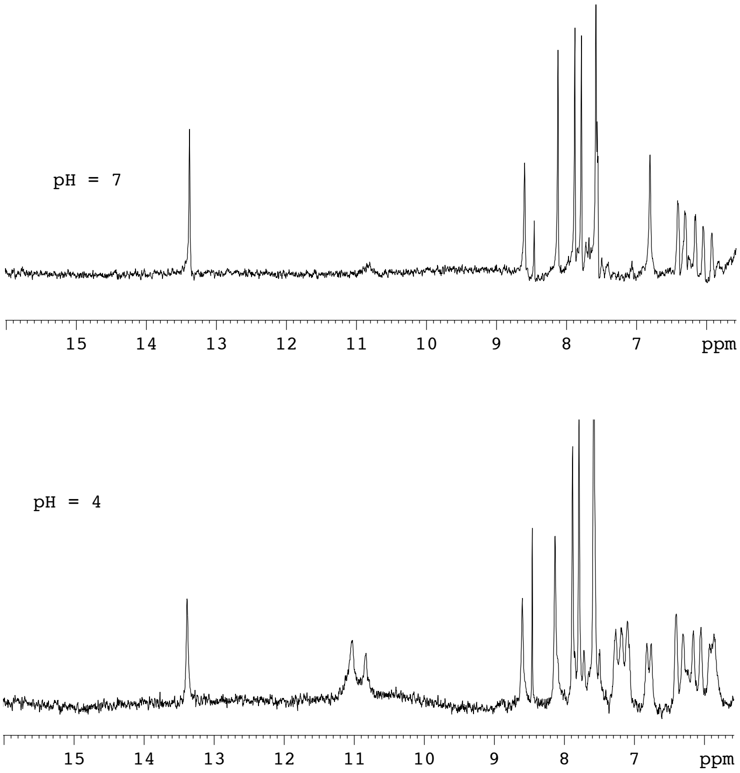

Figure S10. NMR spectra of d<pTGCTTTGCTT> in H_2O/D_2O 9:1 in 25 mM phosphate buffer, 100 mM NaCl T= 5ºC. Top) pH 7.0 Bottom) pH 4.0. 0.5 mM oligonucleotide concentration.

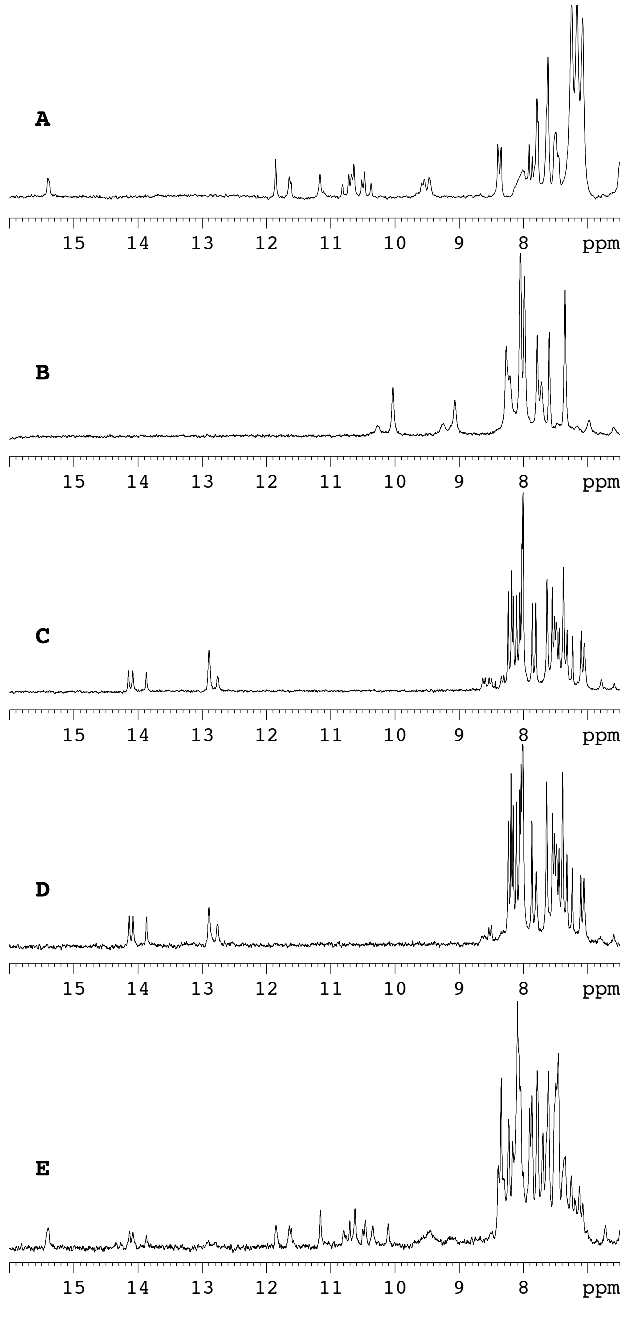

Figure S11. Duplex competition experiments. NMR spectra of: (A) d(TCGTTTCGT) at pH 4.5, T=5ºC (100 μM oligonucleotide concentration, 25 mM phosphate buffer, 100 mM NaCl); (B) Complementary strand d(AGCAAAGCA) at pH 4.5, T=5ºC (100 μM oligonucleotide concentration , 25 mM phosphate buffer, 100 mM NaCl); (C, D and E) Equimolar mixture of d(TCGTTTCGT) and d(AGCAAAGCA), T=5ºC, 100 μM oligonucleotide concentration, 25 mM phosphate buffer, 100 mM NaCl at pH 7, 5 and 4.5, respectively.
